# Exploring the Fecal Microbial Composition and Metagenomic Functional Capacities Associated With Feed Efficiency in Commercial DLY Pigs

**DOI:** 10.3389/fmicb.2019.00052

**Published:** 2019-01-29

**Authors:** Jianping Quan, Gengyuan Cai, Ming Yang, Zhonghua Zeng, Rongrong Ding, Xingwang Wang, Zhanwei Zhuang, Shenping Zhou, Shaoyun Li, Huaqiang Yang, Zicong Li, Enqin Zheng, Wen Huang, Jie Yang, Zhenfang Wu

**Affiliations:** ^1^College of Animal Science and National Engineering Research Center for Breeding Swine Industry, South China Agricultural University, Guangzhou, China; ^2^National Engineering Research Center for Breeding Swine Industry, Guangdong Wens Foodstuffs Group Co., Ltd., Guangzhou, China; ^3^Department of Animal Science, Michigan State University, East Lansing, MI, United States

**Keywords:** DLY pigs, feed efficiency, gut microbiota, 16S rRNA gene, metagenome sequencing

## Abstract

Gut microbiota has indispensable roles in nutrient digestion and energy harvesting, especially in processing the indigestible components of dietary polysaccharides. Searching for the microbial taxa and functional capacity of the gut microbiome associated with feed efficiency (FE) can provide important knowledge to increase profitability and sustainability of the swine industry. In the current study, we performed a comparative analysis of the fecal microbiota in 50 commercial Duroc × (Landrace × Yorkshire) (DLY) pigs with polarizing FE using 16S rRNA gene sequencing and shotgun metagenomic sequencing. There was a different microbial community structure in the fecal microbiota of pigs with different FE. Random forest analysis identified 24 operational taxonomic units (OTUs) as potential biomarkers to improve swine FE. Multiple comparison analysis detected 8 OTUs with a significant difference or tendency toward a difference between high- and low-FE pigs (*P* < 0.01, *q* < 0.1). The high-FE pigs had a greater abundance of OTUs that were from the Lachnospiraceae and Prevotellaceae families and the *Escherichia*-*Shigella* and *Streptococcus* genera than low-FE pigs. A sub-species *Streptococcus gallolyticus* subsp. *gallolyticus* could be an important candidate for improving FE. The functional capacity analysis found 18 KEGG pathways and CAZy EC activities that were different between high- and low-FE pigs. The fecal microbiota in high FE pigs have greater functional capacity to degrade dietary cellulose, polysaccharides, and protein and may have a greater abundance of microbes that can promote intestinal health. These results provided insights for improving porcine FE through modulating the gut microbiome.

## Introduction

Feed cost accounts for nearly 70% of the total cost in pig production (Teagasc, 2015). Therefore, improving feed efficiency (FE) of the pig will reduce feeding expense and increase profitability while also reducing the environmental impact of pig production (Rotz, 2004). In the commercial pig population, especially in Duroc × (Landrace × Yorkshire) (DLY) pigs, the improvement in FE will bring obvious benefits. The FE can be measured by using residual feed intake (RFI) or the feed conversion ratio (FCR). The FCR is calculated as the feed intake divided by the body weight gained. In other words, the high-FCR individuals are less efficient at converting feed into body weight than the low-FCR individuals. The FE in this study was measured by FCR.

In recent years, analyzing the microbiota of breeding animals has gained interest because it allows for the prediction of the potential function and associated metabolites of such communities, which are believed to impact all aspects of host physiology including nutrient processing, energy harvesting, and animal performance (Hiergeist et al., 2015; Xiao et al., 2016; Ferrario et al., 2017; Fouhse et al., 2017; Tan et al., 2017). Previous studies have revealed a possible link between the intestinal microbiota and FE in pigs; e.g., Tan et al. (2018) discovered that in Landrace pigs, the high-FCR pigs had a greater abundance of *Lactobacillus* and *Streptococcus* than the low-FCR pigs. In Large White × Landrace pigs, there was a greater abundance of *Christensenellaceae*, *Oscillibacter*, and *Cellulosilyticum* in the gastrointestinal tract of high-FE pigs (McCormack et al., 2017). In Duroc pigs, Yang et al. (2016) identified 31 operational taxonomic units (OTUs) showing potential associations with FE. Interestingly, these studies also imply that in different breeds of pigs, there may be differences in microbial composition and advantage species. Xiao et al. (2017) also indicated that breed-specific bacteria in swine intestinal tract may exist, even when pigs were treated with the same diet, farm conditions, and management methods.

There are few studies that focus on the association between microbial composition and functional capacity in regards to FE in DLY pigs. For DLY pigs, which are the largest population in the world porcine industry, understanding the relationships between the intestinal tract and host FE performance is meaningful. In our previous studies, we found that DLY pigs with contrasting FE have 11, 55, and 55 OTUs that were different among ileum, cecum and colon (Quan et al., 2018). The functional predictive analysis suggested that the microbial fermentation in cecum and colon may play important roles in improving porcine FE. However, due to the limitations of the research strategy, we have not been able to annotate the microbial gene into more functional database and get more detailed microbial classification differences between high- and low- FE pigs. In this study, we used 16S rRNA gene sequencing and high-throughput metagenomic sequencing to investigate whether the microbiota composition and potential functionality of the intestinal microbiota are linked with FE.

## Materials and Methods

### Animals and Sample Collection

This study was conducted according to the protocols approved by the Animal Care and Use Committee (ACUC) of the South China Agricultural University (SCAU) (approval number SCAU#0017). In an experimental pig farm (Guangdong, Yunfu, Southern China), a total of 226 normal weaning (28-day-old) commercial DLY female pigs were randomly raised in a fattening house comprised of 30 pens, each housing 6–8 pigs. All of the pigs that were analyzed in this study were selected from populations with similar genetic backgrounds and were the same gender. During the fattening stage, the pigs were raised with the same customized diet in man-controlled farm conditions and similar management conditions. The customized corn-soybean feed (free of probiotics and antibiotics) contained 16% crude protein, 3100 kJ of digestible energy and 0.78% lysine. The diet was available *ad libitum* from an automatic feeding trough, Osborne’s FIRE (Feed Intake Recording Equipment) System (Osborne Industries inc, Osborne, Kansas), which can separately record daily feed intake and daily body weight gain of each pig. Water was available *ad libitum* from nipple drinkers. During the whole experiment, any pigs treated with antibiotics were removed from the study. The FCR values of all pigs were calculated at 140 days of age. After the FCR value ranking of each pig, the 25 pigs with the lowest FCR (highest FE) and the 25 pigs with the highest FCR (lowest FE) were selected for this study. The fecal samples of 50 sows were collected following rectal stimulation and were transferred immediately to liquid nitrogen for temporary storage. Then, the samples were sent to the laboratory where they were stored at −80°C until analysis. We further chose six fecal samples for metagenomic sequencing. These six pigs included three individuals from the high-FE group and their full siblings from the low-FE group.

### DNA Extraction, PCR Amplification, and 16S rRNA Gene Sequencing

Fecal DNA was extracted using a Soil Genome^TM^ DNA Isolation Kit (Qiagen, Düsseldorf, Germany) in accordance with the manufacturer’s instructions. DNA concentration and quality were measured using UV-Vis spectrophotometry (NanoDrop 2000, Waltham, MA, United States) and agarose gel electrophoresis. The DNA obtained from each sample was diluted to 1 ng/μL with sterile water. Amplification of the V4–V5 hypervariable region of the bacterial 16S rRNA gene was performed using universal primers, where the reverse primer contained a 6-bp error-correcting barcode unique to each sample (515f: 5′-GTGCCAGCMGCCGCGGTAA-3′, 907r: 5′-CCGTCAATTCCTTTGAGTTT-3′). Amplification was performed using an initial denaturation at 98°C for 1 min followed by 30 cycles of denaturation at 98°C for 10 s, annealing at 50°C for 30 s, elongation at 72°C for 30 s, and a final step at 72°C for 5 min. All PCR reactions were carried out using Phusion^®^ High-Fidelity PCR Master Mix (NEB, Ipswich, MA, United States). PCR products were run in an electrophoresis chamber on a 2% agarose gel to confirm the successful amplification of the target gene. DNA bands of 400–450 bp, corresponding to the 16S rRNA gene amplicon, were excised and purified using the GeneJET Gel Extraction Kit (Thermo Fisher Scientific, Waltham, MA, United States) according to the manufacturer’s instructions. Purified amplicons were used for library preparation and pyrosequencing. Sequencing libraries were generated using NEB Next^®^ Ultra^TM^ DNA Library Prep Kit for Illumina (NEB, Ipswich, MA, United States), following the manufacturer’s recommendations, and index codes were added. A Qubit@ 2.0 Fluorometer (Thermo Fisher Scientific, Waltham, MA, United States) and Agilent Bioanalyzer 2100 system were used to assess the quality of the library. Pyrosequencing was performed on the Illumina HiSeq 2 × 250 platform (Illumina, San Diego, CA, United States). The 16S rRNA gene sequence data have been deposited in the NCBI SRA database with an accession number of SUB4418365.

### Processing of Sequencing Data

Sequencing reads were assigned to each sample, based on unique barcodes, and truncated by cutting off the barcode and primer sequence. The original DNA fragments were merged into tags using FLASH (v1.2.7) (Magoc and Salzberg, 2011). Quality filtering of the raw tags was performed under specific filtering conditions to generate high-quality clean tags according to the QIIME (v1.9.1) quality-controlled process (Caporaso et al., 2010). To generate effective tags, the chimeric sequences were removed from clean tags using the UCHIME algorithm based on the reference database (Gold database) (Haas et al., 2011). After selecting representative species for each OTU, each of the remaining sequences was assigned to an OTU when at least 97% threshold identity was obtained using UPARSE software (v7.0.1) (Edgar, 2013). The taxonomy of each OTU representative sequence was assigned for further annotation using the RDP Classifier algorithm^1^ (Wang et al., 2007) against the SILVA ribosomal RNA gene database. Subsequent analyses were performed based on the OTU information. A Venn diagram was generated using the *VennDiagram* R package to show shared and unique OTUs between high- and low-FE pigs.

In this study, we used mothur software (v.1.30.1) to calculate the community alpha diversity indices, including Chao1 and ACE indices, which estimate community richness, and Shannon and Simpson indices, which estimate community diversity (Schloss et al., 2011). A significant difference in alpha diversity between high- and low-FE groups was determined using the Mann–Whitney *U*-test. Moreover, we also calculated the community pan-OTU number and Good’s coverage index to evaluate sample size and the sequencing depth. Principal component analysis (PCA) was determined to evaluate the community structure similarity between the samples in the high- and low-FE groups. Significant differences in beta-diversity across opposite FE groups were evaluated using permutational multivariate analysis of variance (PERMANOVA) with 10^4^ permutations. In addition, the effects of pen information, initial weight and final weight on variance of sample microbial community composition were evaluated by PERMANOVA analyses (Anderson, 2001; Anderson and Walsh, 2013). Bacterial taxonomic distributions of sample communities were visualized using the *ggplot2* R package. In subsequent analyses, taxa occurring in less than three samples with a relative abundance less than 0.01% of the total community were removed. To test whether microbial community composition can predict feed conversion, we trained a random forest model at the OTU level on all samples based on a random sampling with replacement (Number of decision trees = 500). We evaluated the performance using 10-fold cross-validation. The cross-validation error curve (average of 5 test sets each) of the 10-fold cross-validation was averaged. The variable importance by mean decrease in accuracy was calculated. The predictive power was scored in a receiver operating characteristic (ROC) analysis. The discriminatory power of OTUs was calculated as the area under the ROC curve (AUC) using the *plotROC* R package.

The comparison of relative abundances of OTUs between high- and low-FE pigs was performed using Welch’s *t*-test in STAMP software (White et al., 2009). The Benjamini–Hochberg False Discovery Rate (FDR) method (*q*-value) was used to correct the multiple comparisons (Benjamini and Hochberg, 1995). The statistical cutoff of the *p*-value <0.05 (Welch-Test) and *q*-value <0.05 (FDR) were set as the significance threshold. The relative abundance of different OTUs between high- and low-FE pigs was visualized by heatmap using *vegan* R package.

### Metagenomic Sequencing and Statistical Analyses

Metagenome sequencing libraries were generated with an insert size of 350 base pairs (bp) for six fecal DNA samples following the manufacturer’s instructions (Illumina, San Diego, CA, United States). The libraries for metagenomic analysis were sequenced on an Illumina HiSeq 2500 platform by an Illumina HiSeq – PE150 strategy. The raw reads were treated to remove reads with low qualities, trim the read sequences and remove adaptors using Readfq software (v8). The metagenomic sequencing data have been deposited in the NCBI SRA database with the accession number SUB4056369. Subsequently, pig genomic DNA sequences were removed by SOAPaligner software (v2.21) (Li et al., 2008).

*De novo* assembly of high quality reads was performed using SOAPdenove software (v2.04) with the parameters -d 1, -M 3, -R, -u, -F. Scaffolds were broken into new scaftigs at their gaps (Luo et al., 2012). Meanwhile, the scaftigs with a length less than 500 bp were removed, and the number of scaftigs ≥500 bp was calculated. The qualified scaftigs were applied to predict the bacterial open reading frames (ORFs) by MetaGeneMark (v2.10) software, and the sequences with lengths less than 100 bp were filtered out (Zhu et al., 2010). CD-HIT software (v4.5.8) was used to exclude the redundant genes from all predicted ORFs to construct a preliminary non-redundant gene catalog (Fu et al., 2012). Subsequently, clean reads of each sample were compared to the preliminary non-redundant gene catalog using SOAPaligner with the parameters of -m 200, -× 400, identity ≥95%. The number of reads was compared for each gene that could be calculated. The genes with a read number ≤2 were removed to obtain a final non-redundant gene catalog (Qin et al., 2012). The genes in the final non-redundant gene catalog were called unigenes. The abundance of a gene was calculated based on the number of reads that aligned to the gene, normalizing by the gene length and the total number of reads aligned to the unigenes (Karlsson et al., 2012). The specific formula for the relative abundance calculation of a gene was *G_k_* = $\frac{r_{k}}{L_{k}}\cdot \frac{1}{\sum_{i=1}^{n}\frac{r_{i}}{L_{i}}}$., here r is the number of reads mapped to a gene and L is the length of gene. Subsequently, we used DIAMOND software (V0.7.9) to compare the unigenes with the Kyoto Encyclopedia of Genes and Genomes (KEGG) gene database to obtain KO annotation information and metabolic pathway information (Buchfink et al., 2015). We compared the unigenes with the Carbohydrate-Active enzymes database (CAZy) to obtain information on species and the functional classification of EC.

To determine the differential abundance of functional features between the high- and low-FE groups, Metastats analysis was applied (White et al., 2009). The Benjamini–Hochberg FDR method (*q*-value) was used to correct the multiple comparisons (Benjamini and Hochberg, 1995). *Z*-scores were calculated to construct a heatmap to demonstrate the relative abundance of the pathways in each group with the formula *z* = (x−μ)/σ, where x is the relative abundance of the pathways in each group, μ is the mean value of the relative abundances of the pathways in all groups, and σ is the standard deviation of the relative abundances.

## Results

### Phenotypic Values of Porcine FCR and Community Composition of Porcine Fecal Microbiota

**FIGURE 1:**
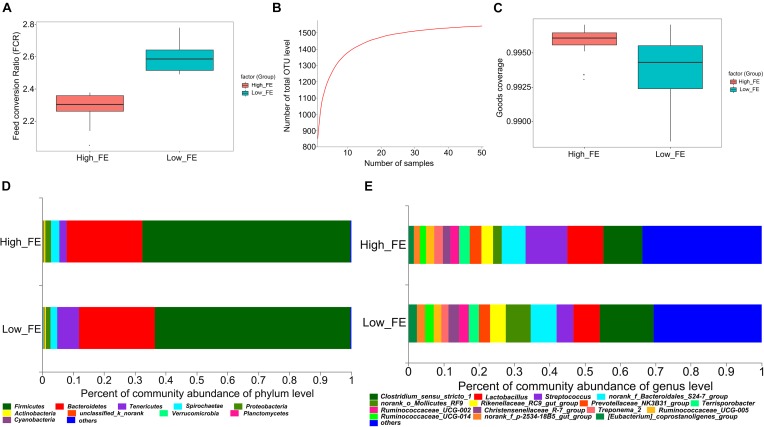
The feed conversion ratio value (FCR), pan OTUs, Good’s coverage and community composition in high- and low-feed-efficiency (FE) pigs. Groups are coded according to the feed efficiency status (High_FE, high feed efficiency; Low_FE, low feed efficiency). **(A)** FCR value in high- and low-FE pigs. **(B)** Pan OTU = sample size. The horizontal axis represents the number of samples. The vertical axis represents the number of OTUs contained in all samples. **(C)** Good’s coverage value in high- and low-FE pigs. **(D)** Community composition at the phylum level in high- and low-FE pigs. **(E)** Community composition at the genus level in high- and low-FE pigs.

All experimental pigs had daily feed intake and daily body weight gain separately recorded during the fattening stage (28-day-old to 140-day-old). The 25 pigs with the highest FE (FCR value: 2.29 ± 0.080) and the 25 pigs with the lowest FE (FCR value: 2.60 ± 0.088) were selected for this study. The FCR value was significantly different between the high- and low-FE groups (*p*-value < 0.001, Figure 1A and Supplementary Table S1).

A total of 50 pigs, which included extreme FCR values, were selected, and 16S rRNA gene sequencing was performed, which generated a total of 3,788,293 DNA sequence reads, aligned into 2,851,748 effective tags after quality control. Based on the 97% sequence similarity, the number of OTU samples ranged from 569 to 1037. The pan-OTU numbers of community would reach saturation when the sample size was greater than thirty (Figure 1B) and the Good’s coverage indices in high- and low-FE groups were greater than 99% (Figure 1C), which indicated a sufficient sample size and adequate sequencing depth for this study. The Venn diagrams show that 1437 OTUs were shared between the high- and low-FE groups. Only 44 and 60 OTUs were unique in the low- and high-FE groups, respectively (Supplementary Figure S1A).

These OTUs were annotated to the phylum, class, order, family and genus classification level. At the phylum level, the high- and low-FE pigs’ microbial community was dominated by Firmicutes (67.47% vs. 63.35%), Bacteroidetes (24.40% vs. 24.68%), Tenericutes (2.43% vs. 7.01%), Spirochaetes (2.66% vs. 2.11%), and Proteobacteria (1.93% vs. 1.49%) (Figure 1D). At the genus level, *Streptococcus* (11.80%), *Clostridium sensu stricto 1* (11.11%), and *Lactobacillus* (10.19%) were the three most abundant genera in the high-FE group; *Clostridium sensu stricto 1* (15.21%), *Lactobacillus* (7.51%), and uncertain genera from Bacteroidales S24-7 (7.40%) were the three most abundant genera in the low-FE group (Figure 1E).

To further investigate microbial composition at the species level, shotgun metagenomic sequencing was performed in six fecal samples from three pairs of full-siblings having the high- and low-FCR phenotypes. The metagenomic sequencing produced a total of 56 Gbp of clean reads after removing low-quality sequences and host genomic DNA sequences. After subsequent assembly, a total of 1.15 million scaftigs with an average length of 1,095 bp and an average N50 length of 1,152 bp were produced. The phylogenetic composition of the fecal microbiota determined by shotgun metagenomic sequencing was similar to the result obtained in the 16S rRNA gene sequencing. Firmicutes, Bacteroidetes, Spirochaetes, and Proteobacteria were the dominant phyla (Supplementary Figure S1B). At the species level, we detected a total of 6,972 bacterial species from all six fecal samples. *Firmicutes bacterium CAG:110*, *Treponema bryantii* and *Bacteroides* sp. *CAG:1060* were the three most abundant species (Supplementary Figure S1C).

### Comparison of Fecal Microbial Community Diversity Between High- and Low-FE Pigs

**FIGURE 2:**
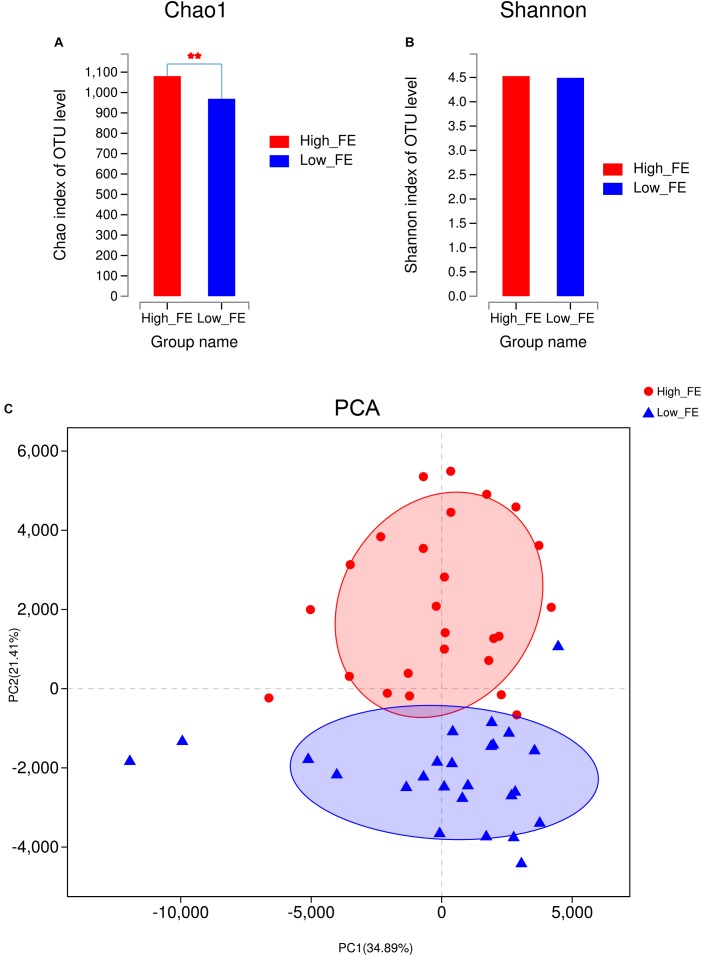
The community diversity between fecal samples from high- and low-FE pigs at the OTU level. **(A)** The Chao1 index in high- and low-FE pigs. **(B)** The Shannon index in high- and low-FE pigs. **(C)** Principal component analysis (PCA) of the fecal microbiota based on OTUs.

To evaluate the alpha-diversity of bacterial communities in high- and low-FE pigs, we compared the community richness indices (Chao1 and ACE) and diversity indices (Shannon and Simpson) of the microbiota in high- and low-FE pigs. We found that high-FE pigs have significantly higher Chao1 and ACE indices than low-FE pigs (*P* < 0.01, Figure 2A and Supplementary Figure S2A). However, the Shannon and Simpson indices were not significantly different between these two groups (Figure 2B and Supplementary Figure S2B). Based on the abundance profiling of the OTU level, PCA analysis showed that most of the samples could be clustered into two groups, which was very consistent with the grouping results according to performance of feed conversion (Figure 2C). A significant dissimilarity in beta-diversity between high- and low-FE groups was observed (PERMANOVA, *p*-value <0.01). Based on the abundance profiling of species level generated by metagenomic sequencing, there were also a clear difference in bacterial composition in the high- and low-FE pigs (Supplementary Figure S2C). In addition, we found that the initial weight and final weight had no significant effect on porcine fecal microbial composition (*p*-value >0.3). The pig pen had a tendency to make effect on microbial composition, but also cannot reach the significant level in our study (*p*-value = 0.077) (Supplementary Table S2).

### Identification of Potential Biomarkers That Could Account for the FE Differences

**FIGURE 3:**
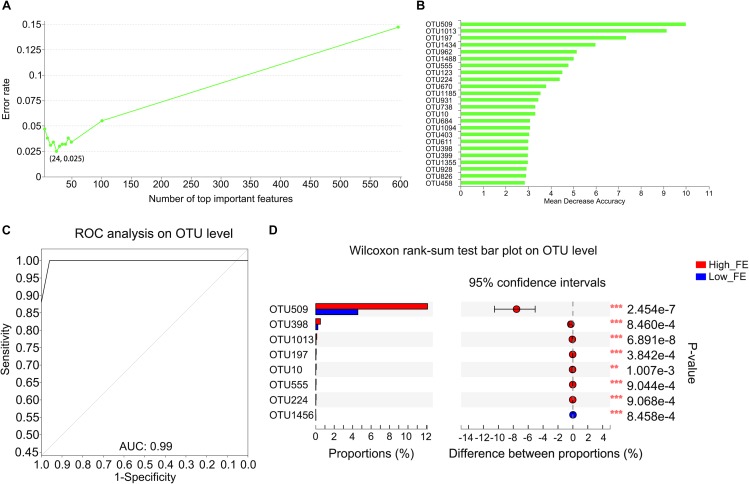
Important biomarkers selected by random forest analysis and the different abundance of OTUs in high- and low-FE pigs. **(A)** Model evaluation by using top important variables. The horizontal axis represents the number of variables ranked by importance. The vertical axis denotes the average prediction error rate using 10-fold cross-validation when using the number of corresponding variables. **(B)** The ordination diagram of variables of importance. The horizontal axis is the measurement standard of variables of importance, and the value is equal to the measurement value of variables of importance/standard deviation. The vertical axis is the variable names sorted by importance. **(C)** ROC of the random forest classifier based on the 24 most important variables. The AUC value is the area under the corresponding curve. When the AUC > 0.5, the AUC value is closer to 1, the diagnostic effect is better. **(D)** The OTUs with a significantly different abundance between high- and low-FE pigs detected by STAMP software.

To determine whether OTUs could serve as biomarkers to classify pigs into high- and low-FE groups and which OTUs play important roles in this process, we constructed a random forest model. The OTU-level random forest model had an error of 0.025 when the number of top important variables was 24 (Figure 3A). The mean decrease in accuracy of the top 24 important variables is shown in Figure 3B. Six OTUs that included the top three important variables (OTU509, OTU1013, and OTU197) for predicting FE were annotated to the genus of *Streptococcus*. Fortunately, based on the existing database information, the most important candidate biomarker (OTU509) can also be annotated to species level, which was *Streptococcus gallolyticus* subsp. *gallolyticus*. Ten OTUs were annotated to the family of Lachnospiraceae (OTU962, OTU555, OTU1185, OTU931, OTU738, OTU684, OTU403, OTU399, OTU928, and OTU458). Two pairs of OTUs were annotated to the families of Erysipelotrichaceae (OTU1434 and OTU826) and Ruminococcaceae (OTU1094 and OTU1355). Four single OTUs were annotated to the families Coriobacteriaceae (OTU123), Peptococcaceae (OTU670), Prevotellaceae (OTU10), and Enterobacteriaceae (OTU398) (Figure 3B and Supplementary Table S3). The area under the ROC curve (AUG) was 0.99 based on the 24 most important variables (Figure 3C).

We further compared the abundance of OTUs between the high- and low-FE pigs using STAMP software with Welch’s *t*-test. We detected only two OTUs (OTU509 and OTU1013) that were significantly different between pigs with low or high FE using *p*-value <0.05 and *q*-value <0.05 as the significance threshold. However, at a threshold of *p*-value <0.01 and *q*-value <0.1, we identified an additional six OTUs with a tendency toward a difference (Figure 3D). The average abundance of these OTUs between the high- and low-FE groups are shown in Supplementary Figure S3. Most of these OTUs were contained in the OTU list that outlined important variables to account for the differences in FE (Supplementary Table S4), except OTU1456, which was annotated to the order Clostridiales.

### Comparison of the Functionality of the Fecal Microbiome in High- and Low-FE Groups Based on Metagenomic Sequencing

**FIGURE 4:**
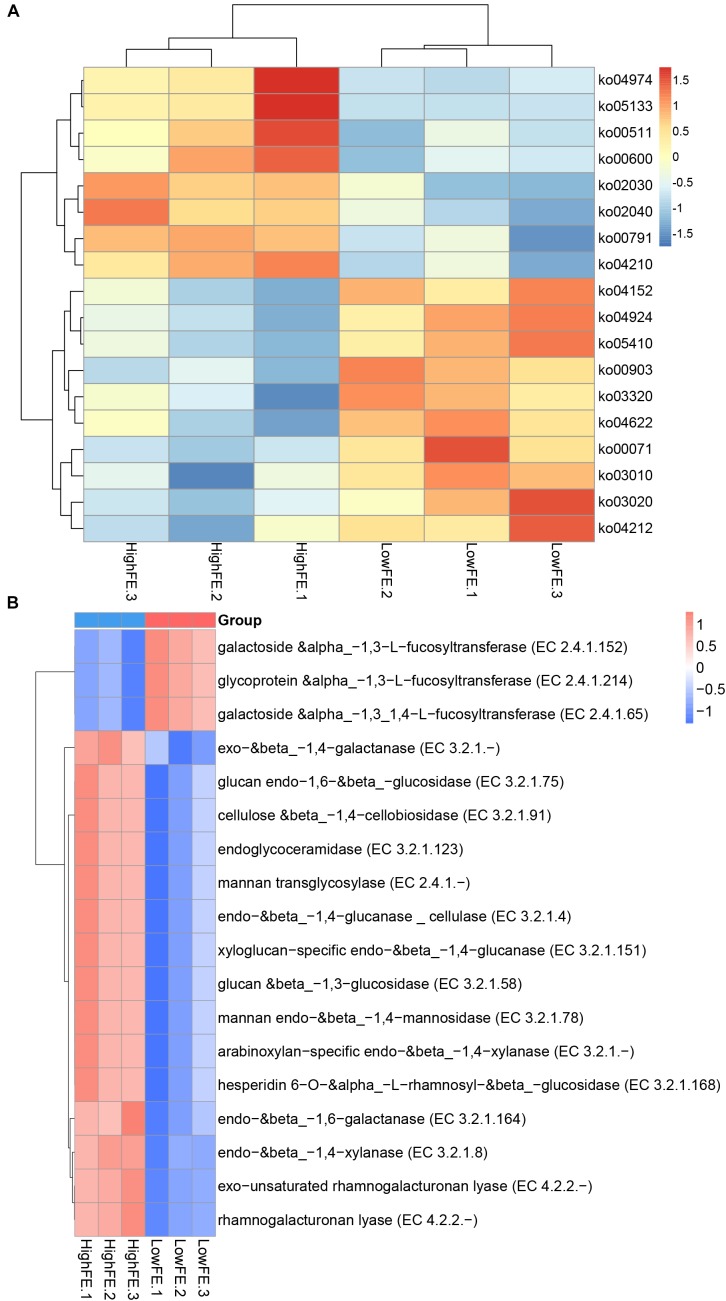
Heatmap of functional capacity profiles showing different enrichment in high- and low-FE pigs by metagenomic sequencing analysis. Samples are coded according to the feed efficiency status (HighFE, high feed efficiency; LowFE, low feed efficiency). For example, HighFE.1 represented the first sample that was collected from high-feed-efficiency pig. **(A)** Heatmap of KEGG pathways showing different enrichments in high- and low-FE pigs. **(B)** Heatmap of CAZy EC activities showing different enrichments in high- and low.

Comparison of the functional capacity of the gut microbiome can help to investigate the metabolic differences between high- and low-FE groups and further indicate the microbes that may affect special nutrient metabolism. The functional capacity was determined according to the annotation of ORFs predicted from the assembled contigs. The predicted genes were then aligned with the KEGG gene database to obtain the KO annotation information from the KEGG database (see section “Materials and Methods”). A total of 1,857,107 ORFs were found with an average length of 616 bp. We identified a total of 352,002 KEGG genes and assigned them into 322 KEGG pathways. Subsequently, we compared the KEGG pathways abundance between the high- and low-FE groups, but no pathways were significantly different at FDR < 0.05. When we relaxed the threshold (*p*-value <0.05 and *q*-value <0.3), 18 pathways showed different enrichment at level 3. Eight pathways were more enriched in high-FE groups, and 10 pathways were more enriched in low-FE groups. The pathways that were enriched in high-FE pigs were associated with protein metabolism (ko04974), lipid metabolism (ko00600), and glycan degradation (ko00511). The pathways enriched in low-FE pigs involved endocrine regulation (ko03320 and ko04924), signal transduction (ko04152), the immune system (ko04622) and cardiovascular diseases (05410) (Figure 4A and Supplementary Table S5).

We further investigated the functional information of genes in the CAZy database; over forty thousand genes were identified and categorized into six CAZy classes. Glycoside hydrolases (GHs), glycosyl transferases (GTs) and carbohydrate-binding modules (CBMs) were the three classes enriched the most in both the high- and low-FE groups (Supplementary Figure S4). When we compared the EC activity abundance between the high- and low-FE groups, we found that 15 EC activities were more abundant in the high-FE groups, and 3 EC activities were enriched in the low-FE groups (*p*-value <0.05, *q*-value <0.05). The higher abundance of EC activities in the high-FE groups involved the degradation of xylan, cellulose and many other polysaccharides. The low-FE pigs have a greater abundance of three kinds of fucosyltransferases than the high-FE pigs (Figure 4B and Supplementary Table S6).

## Discussion

Metagenomic approaches based on high-throughput sequencing methods have rapidly facilitated the compositional and functional study of the gut microbiota in recent years (Fraher et al., 2012; Weinstock, 2012). Based on these high-throughput sequencing methods, many previous studies had revealed potential microbial biomarkers for improving FE in multiple breeds of pigs (McCormack et al., 2017; Tan et al., 2017, 2018; Yang et al., 2017; Quan et al., 2018). However, this study is one of the first to combine the technology of 16S rRNA gene sequencing and shotgun metagenomic sequencing to analyze the fecal microbial composition and function in commercial DLY pigs with high and low FE. All experimental pigs were selected from populations with similar genetic backgrounds. They were the same gender and were subjected to the same environmental, nutritional and management conditions to minimize the variability in FE due to genetic, gender, and external factors. Even in this well-controlled environment, there was still polarization in the FE of the experimental pig population. The difference in the intestinal microbiota in the experimental pigs was partly contributed to this phenomenon, which was suggested by previous studies (Nicholson et al., 2012; Parks et al., 2013; Yang et al., 2014). Although the experimental pig population and the number of sequencing samples is not particularly large in this study, the obvious FE variations reflect the real phenomenon in the pig industry. The pan-OTU number and the Good’s coverage indices in the sequencing samples showed sufficient sampling of the population and adequate depth to investigate different bacterial species in the high- and low-FE pigs. The different bacterial species that were involved in feed nutrient processing and energy harvesting in high- and low-FE DLY pigs could be considered potential microbial biomarkers for FE.

When looking at the fecal microbiota composition, consistent with previous findings in pigs, the core phyla within the fecal microbiota were dominated by Firmicutes and Bacteroidetes (Yang et al., 2016; Xiao et al., 2017). Bacteroidetes have an important role in degrading indigestible dietary polysaccharides into short-chain fatty acids that can be reabsorbed by the host (Becker et al., 2014). Firmicutes were also thought to play a vital role in the energy harvest of mice (Turnbaugh et al., 2006). This finding may indicate that the dominant core phyla maintain a balance and can ensure the stability of intestinal function during the growth process in pigs. However, in our present study, we did not observe differences in *Bacteroides* and Firmicutes in high- and low-FE pigs. These findings differed with the results in pigs that showed an increase in Firmicutes in high fatness compared with low fatness subjects (Yang et al., 2016). Furthermore, when considering the annotation result at the genera level of the swine fecal microbiota, many studies had different classification compositions. In Yang et al. (2016) study, *Prevotella*, *Lactobacillus*, and *Treponema* were the three most abundant genera in Duroc pigs. *Prevotella*, *Streptococcus*, and *SMB53* were the three most abundant genera in Hampshire pigs, and *Clostridium*, *SMB53*, and *Streptococcus* were the three most abundant genera in Landrace and Yorkshire pigs in Xiao et al. (2017) study. This result suggested a special composition of the intestinal microbial community at the genus level, which may be due to differences in the breed, age, feed, and husbandry of pigs.

When we compared the bacterial community composition between the high- and low-FE pigs, we found that the community structure was significantly different (Figure 2A,C). The community of high-FE pigs had more richness and similar diversity to that of low-FE pigs. This finding suggested that the difference in FE is not due to the presence of specific bacteria in high-FE pigs but to the larger number of certain bacteria. These differences may come from colonized difference in the early life of mammalian, whose gut microbiome were thought to be at least partially shared by their parents, and it is relatively stable to perturbation once a dense microbial population is established (Antonopoulos et al., 2009; Snijders et al., 2016). However, no study has confirmed the causality between the microbial difference of offspring and their parents in pigs. In the present study, since the dams of our experimental pigs could not be fully tracked, we could not conclude that mother animals would cause a bias between high- and low FE pigs. In addition, gut microbiota composition may also be influenced by environmental factor (Yang et al., 2017), such as pig pen. However, in our study, the pig pen did not have a significant effect on porcine fecal microbial composition, but had a tendency to take a significant effect (*p*-value = 0.077) (Supplementary Table S2). In the current study, the random forest analysis showed that many OTUs played important roles in varying FE (Figure 3B). According to the annotation information from these OTUs, bacteria belonging to the genus *Streptococcus* and the families Lachnospiraceae, Erysipelotrichaceae, Ruminococcaceae, Coriobacteriaceae, Peptococcaceae, Prevotellaceae, and Enterobacteriaceae may be important candidates to improve swine FE. Furthermore, the OTUs that were enriched in high-FE pigs were mainly found in *Streptococcus*, *Escherichia*-*Shigella*, and Prevotellaceae *NK3B31* and the family Lachnospiraceae (Figure 3D and Supplementary Table S4).

A previous study suggested that Lachnospiraceae was associated with human obesity (Cho et al., 2012). Kameyama and Itoh (2014) reported that a bacterial strain of Lachnospiraceae can induce obesity in mice. Many members of Lachnospiraceae can produce short-chain fatty acids (SCFAs) by fermenting dietary polysaccharide (Pryde et al., 2002). The SCFAs were linked to a reduced risk of developing gastrointestinal disorders, cancer and cardiovascular disease and promote human obesity (Wong et al., 2006; Cho et al., 2012). Therefore, we hypothesized that the Lachnospiraceae might improve porcine FE by maintaining the gut in a healthy state to increase its absorptive capacity. Prevotellaceae is reported to relate to several diseases, such as asthmatic airway inflammation and arthritis, and associate with mucin degradation (Brinkman et al., 2011; Scher et al., 2013). Several members of Prevotellaceae were well-known succinate producer and can improve glucose homeostasis through activation of intestinal gluconeogenesis (De Vadder et al., 2016). A recent study reported that the succinate level was associated with carbohydrate metabolism and energy production (Serena et al., 2018). This study indicated that Prevotellaceae may increase FE in pigs by promoting host health or energy metabolism. A study in dairy calves suggested that SCFA concentration and carbohydrate utilization were significantly correlated with *Escherichia-Shigella* (Song et al., 2018). *Streptococcus* has been generally considered a health-promoting microbe for its roles in modulating human health (Kleerebezem and Vaughan, 2009). Many species belonging to *Streptococcus* were associated with carbohydrate fermentation, starch hydrolysis and the production of glucan from sucrose (Facklam, 1972; Nelms et al., 1995). In this study, *Streptococcus gallolyticus* subsp. *gallolyticus* (annotated by OTU509) was considered an important candidate that might be used for improving porcine FE. Most strains of this species can ferment mannitol, trehalose, and inulin and can produce acid from starch and glycogen (Schlegel et al., 2003). These finding suggested that high-FE pigs are likely to have a greater abundance of intestinal microbes that can promote host intestinal health or degrade dietary carbohydrates. Therefore, high-FE pigs might have a greater ability to utilize feed and better intestinal health than low-FE pigs.

We also performed functional capacities analyses. Although we did not identify any core metabolic pathways at the *q*-value <0.05 level, some pathways showed a trend toward difference in high- and low-FE pigs. The pathways associated with protein metabolism (ko04974), lipid metabolism (ko00600), and glycan degradation (ko00511) were enriched in high-FE pigs. The higher abundance of protein metabolism and glycan degradation pathways in the high-FE groups had been reported in previous studies (Li and Guan, 2017; Yang et al., 2017). The experimental pigs in this study were fed with a fiber-enriched and high-protein diet. Therefore, the fecal microbiota may be more competent in terms of utilizing the diet protein. It is interesting that the fecal microbes of high-FE pigs have relatively more pathways of lipid metabolism, and it was believed that most of digestion and absorption occur in the small intestine (Rudd, 2012; Voet et al., 2013). We confirmed whether the fecal microbes have a compensatory metabolism function for unmetabolized lipid. Analysis of the microbial gene functional annotation in the CAZy database revealed expected results. In high-FE groups, the EC activities included the degradation of xylan, cellulose and many other polysaccharides. These functional results were consistent with the previous hypothesis that the high-FE pigs might have a greater ability to utilize dairy protein and carbohydrate than low-FE pigs.

## Conclusion

In conclusion, there was a different microbial community structure in the fecal microbiota of pigs with different FE. We detected 24 OTUs that can serve as potential biomarkers to improve swine FE. Eight OTUs were significantly different or had a trend toward difference in the high- and low-FE pigs. The high-FE pigs had a greater abundance of OTUs in the families Lachnospiraceae and Prevotellaceae and in the genera *Escherichia*-*Shigella* and *Streptococcus* compared to low-FE pigs. *Streptococcus gallolyticus* subsp. *gallolyticus* could be an important candidate microbe for improving FE. We detected 18 KEGG pathways and CAZy EC activities that were different between high- and low-FE pigs. We found that the fecal microbiota in high-FE pigs have a greater capacity to degrade dietary cellulose, polysaccharide, and protein and may have a greater abundance of microbes to promote intestinal health. These findings should improve our understanding of the differences in the fecal microbial composition between high- and low-FE commercial pigs and provide important candidate microbes that can potentially use for improving porcine FE.

## Author Contributions

JY and ZW conceived and designed the experiments. JQ, MY, ZhoZ, RD, XW, ZhaZ, SZ, SL, HY, ZL, and EZ performed the experiments. JQ and JY analyzed the data. MY, GC, RD, XW, JY, EZ, and JQ collected the samples and recorded the phenotypes. GC and MY contributed the materials. JQ wrote the manuscript. JY, WH, and ZW revised the manuscript. All authors reviewed and approved the final manuscript.

## Conflict of Interest Statement

The authors declare that the research was conducted in the absence of any commercial or financial relationships that could be construed as a potential conflict of interest.

## Footnotes

1 http://rdp.cme.msu.edu/
