# Supplementary material for: Exploring the Fecal Microbial Composition and Metagenomic Functional Capacities Associated With Feed Efficiency in Commercial DLY Pigs
Source: Front Microbiol. 2019 Jan 29;10:52. doi: 10.3389/fmicb.2019.00052 (PMC6361760; doi:10.3389/fmicb.2019.00052)
Supplement: Supplementary file 1 [file Data_Sheet_1.doc]

**Exploring the Fecal Microbial Composition and Metagenomic Functional Capacities Associated with Feed Efficiency in Commercial DLY Pigs**

**Jianping Quan^1,$^, Gengyuan Cai^1,2,$^, MingYang^2^ ,** **Zhonghua Zeng^1^, Rongrong Ding^1^, Xingwang Wang^1^, Zhanwei Zhuang^1^,** **Shenping Zhou^1^,** **Shaoyun Li^1^, Huaqiang Yang^1^, Zicong Li^1^, Enqin Zheng^1^, Wen Huang^3^, JieYang^1,*^, Zhenfang Wu^1,*^**

^1^College of Animal Science and National Engineering Research Center for Breeding Swine Industry, South China Agricultural University, Guangdong, P.R. China.

^2^National Engineering Research Center for Breeding Swine Industry, Guangdong Wens Foodstuffs Co., Ltd, Guangdong, P.R. China.

^3^Department of Animal Science, Michigan State University, East Lansing, MI, United States.

*** Correspondence:**Zhenfang Wu
[wzfeamil@163.com](mailto:wzfeamil@163.com)

Jie Yang
[jieyang2012@hotmail.com](mailto:jieyang2012@hotmail.com)

^$^These authors contributed equally to this work

**Keywords: DLY pigs, feed efficiency, gut microbiota, 16S rRNA gene,** **metagenome sequencing**

**Supplementary Figures**


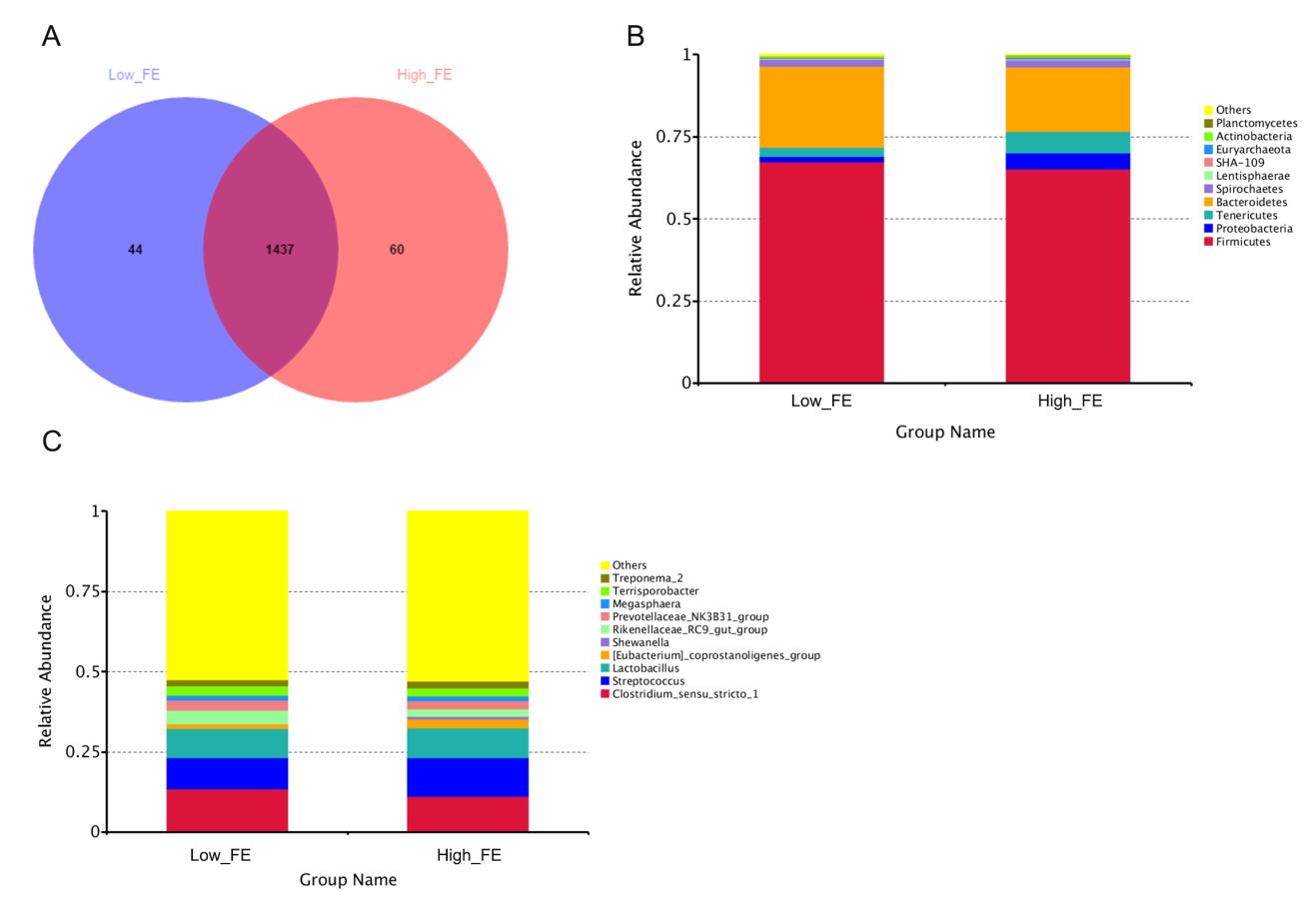


**Figure S1 (**A) The shared and unique OTUs number between high- and low- FE groups. (B) Microbial composition at the phylum level for high- and low- FE groups.

(C) Microbial composition at the genus level for high- and low- FE groups.


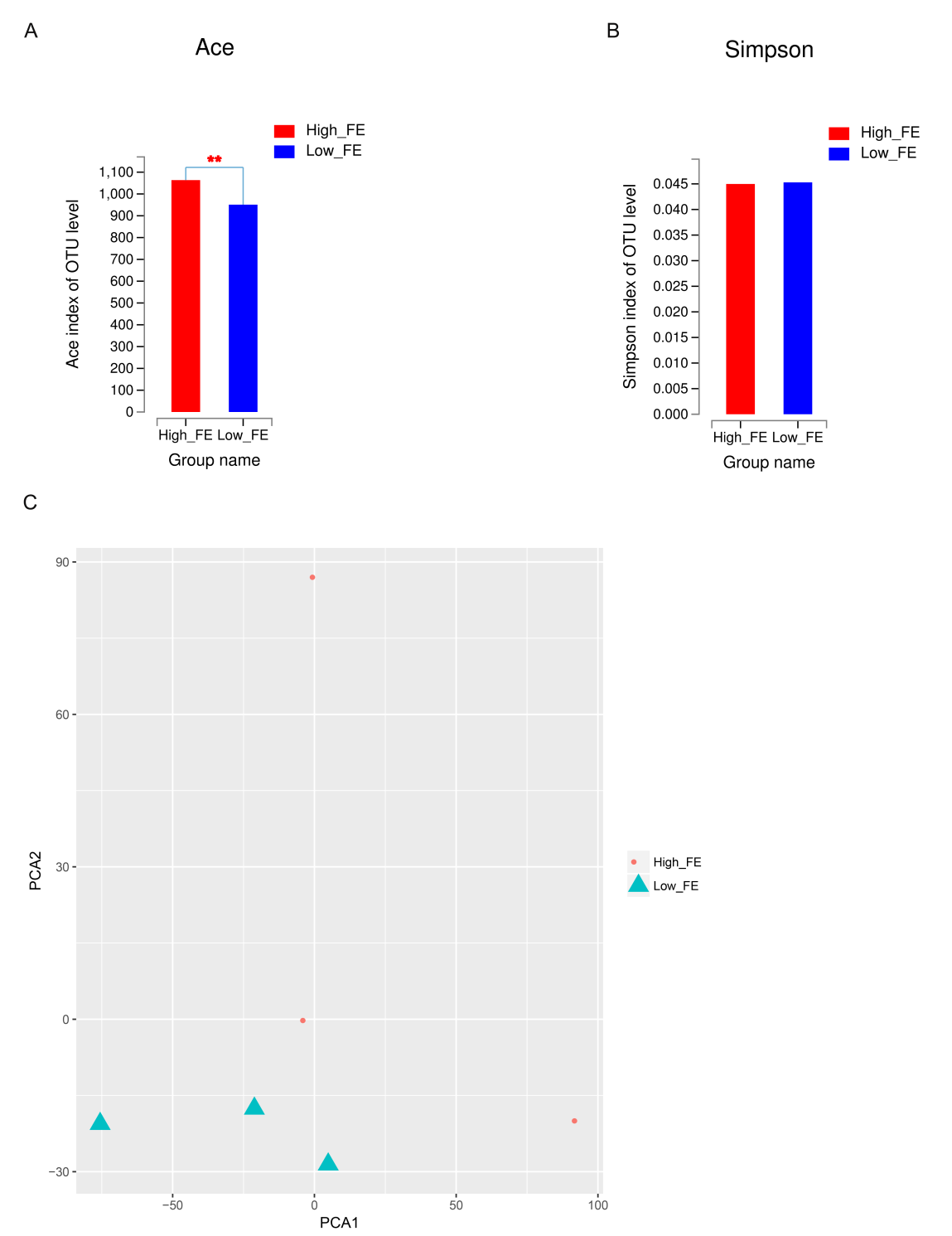


**Figure S2** (A) The ACE index in high- and low-FE pigs. (B) The Simpson index in high- and low-FE pigs. (C) Principal component analysis (PCA) of the fecal microbiota based on the abundance profiling of species level generated by metagenomic sequencing.


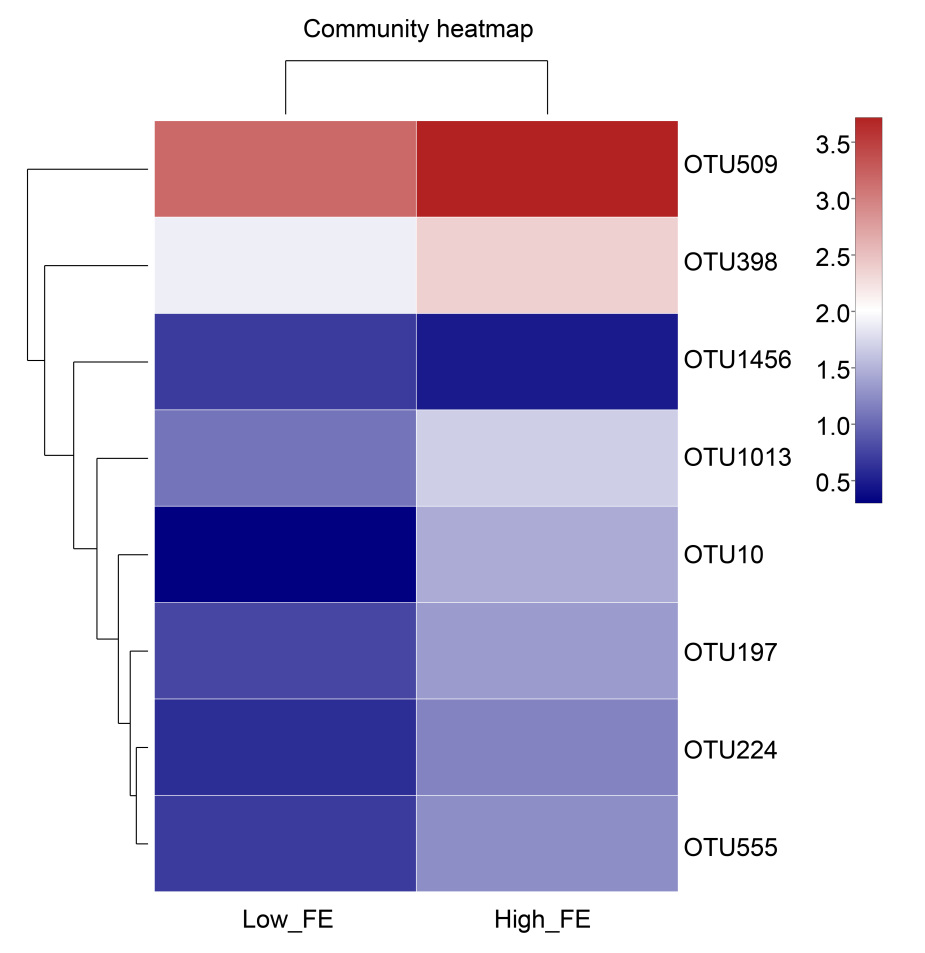


**Figure S3** The average abundance heatmap of different OTUs between high- and Low-FE groups.


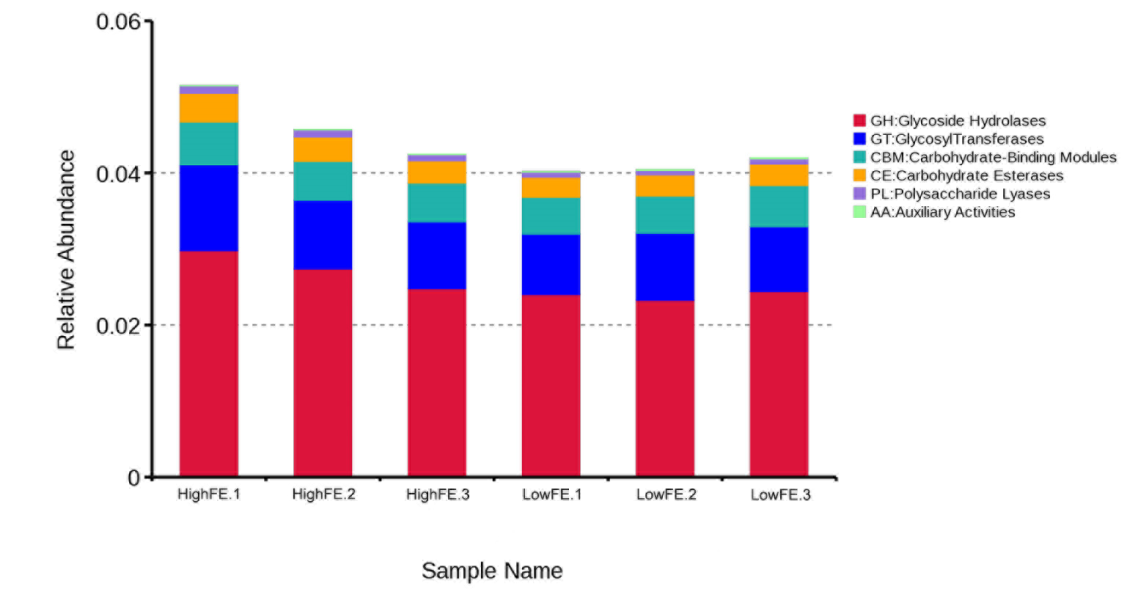


**Figure S4** Functional composition of genes in the CAZy database for high- and low-FE groups.

**Supplementary Tables**

**Table S1** The phenotype information of pigs from High- and Low-FE groups.

| Samples | Groups | FCR_value | Pen_number | Initial_weight | Final_weight |
| --- | --- | --- | --- | --- | --- |
| FecH_1 | High_FE | 2.293 | 9 | 29.6 | 90 |
| FecH_2 | High_FE | 2.051 | 1 | 28 | 92 |
| FecH_3 | High_FE | 2.176 | 13 | 31.1 | 95.2 |
| FecH_4 | High_FE | 2.289 | 13 | 30 | 90.7 |
| FecH_5 | High_FE | 2.284 | 13 | 30 | 90.8 |
| FecH_6 | High_FE | 2.327 | 13 | 30.1 | 89.5 |
| FecH_7 | High_FE | 2.373 | 7 | 28.9 | 87 |
| FecH_8 | High_FE | 2.23 | 1 | 31.6 | 95.9 |
| FecH_9 | High_FE | 2.373 | 26 | 28 | 86 |
| FecH_10 | High_FE | 2.304 | 21 | 31 | 91.7 |
| FecH_11 | High_FE | 2.366 | 17 | 29.9 | 88.7 |
| FecH_12 | High_FE | 2.378 | 7 | 29.3 | 88.2 |
| FecH_13 | High_FE | 2.359 | 25 | 28.8 | 87.3 |
| FecH_14 | High_FE | 2.245 | 15 | 29 | 97 |
| FecH_15 | High_FE | 2.277 | 23 | 31.8 | 88.9 |
| FecH_16 | High_FE | 2.28 | 23 | 29.2 | 86.8 |
| FecH_17 | High_FE | 2.331 | 3 | 31 | 93.6 |
| FecH_18 | High_FE | 2.362 | 11 | 29.8 | 89.6 |
| FecH_19 | High_FE | 2.349 | 26 | 32 | 95 |
| FecH_20 | High_FE | 2.324 | 19 | 30.3 | 88 |
| FecH_21 | High_FE | 2.332 | 15 | 30 | 86.8 |
| FecH_22 | High_FE | 2.367 | 3 | 29.7 | 89.9 |
| FecH_23 | High_FE | 2.14 | 17 | 29.9 | 94.7 |
| FecH_24 | High_FE | 2.243 | 11 | 29.9 | 89 |
| FecH_25 | High_FE | 2.262 | 23 | 29.8 | 89.6 |
| FecL_1 | Low_FE | 2.78 | 25 | 30.4 | 92.5 |
| FecL_2 | Low_FE | 2.606 | 25 | 29.7 | 93.2 |
| FecL_3 | Low_FE | 2.639 | 15 | 29.8 | 89.4 |
| FecL_4 | Low_FE | 2.564 | 19 | 31.2 | 87.2 |
| FecL_5 | Low_FE | 2.491 | 27 | 29.3 | 85.4 |
| FecL_6 | Low_FE | 2.515 | 1 | 34 | 85.6 |
| FecL_7 | Low_FE | 2.563 | 19 | 30 | 94.3 |
| FecL_8 | Low_FE | 2.506 | 27 | 29.9 | 87.4 |
| FecL_9 | Low_FE | 2.618 | 15 | 30.6 | 95.2 |
| FecL_10 | Low_FE | 2.669 | 21 | 29.4 | 88.7 |
| FecL_11 | Low_FE | 2.652 | 9 | 31.5 | 87.2 |
| FecL_12 | Low_FE | 2.642 | 21 | 30.5 | 87.3 |
| FecL_13 | Low_FE | 2.595 | 17 | 32 | 95.6 |
| FecL_14 | Low_FE | 2.579 | 11 | 32.7 | 88.9 |
| FecL_15 | Low_FE | 2.586 | 21 | 30 | 87.6 |
| FecL_16 | Low_FE | 2.558 | 3 | 32.8 | 96.5 |
| FecL_17 | Low_FE | 2.756 | 23 | 28 | 87.5 |
| FecL_18 | Low_FE | 2.514 | 3 | 29.8 | 92.4 |
| FecL_19 | Low_FE | 2.775 | 26 | 28.2 | 91.8 |
| FecL_20 | Low_FE | 2.593 | 13 | 30.1 | 93.4 |
| FecL_21 | Low_FE | 2.7 | 26 | 28.9 | 87.6 |
| FecL_22 | Low_FE | 2.5 | 5 | 31.8 | 87.4 |
| FecL_23 | Low_FE | 2.502 | 23 | 30.4 | 88.1 |
| FecL_24 | Low_FE | 2.496 | 11 | 29.3 | 93.5 |
| FecL_25 | Low_FE | 2.515 | 5 | 27.9 | 85.6 |

**Table S2** The pen, initial weight and final weight effects on porcine fecal microbial composition.

| Characteristics | SumOfSqs | F.Model | R^2^ | p-value |
| --- | --- | --- | --- | --- |
| Pen | 0.1771 | 1.5238 | 0.02827 | 0.077 |
| Initial_wight | 0.1239 | 1.0656 | 0.01977 | 0.380 |
| Final_weight | 0.1254 | 1.0789 | 0.02002 | 0.377 |

**Table S3** The top 24 important OTUs and their annotation information at family and genus levels

| OUT_ID | Mean decrease accuracy | Family | Genus |
| --- | --- | --- | --- |
| OTU509 | 9.993 | *f__Streptococcaceae* | *g__Streptococcus* |
| OTU1013 | 9.144 | *f__Streptococcaceae* | *g__Streptococcus* |
| OTU197 | 7.334 | *f__Streptococcaceae* | *g__Streptococcus* |
| OTU1434 | 5.985 | *f__Erysipelotrichaceae* | *g__Holdemanella* |
| OTU962 | 5.144 | *f__Lachnospiraceae* | *g__Blautia* |
| OTU1488 | 5.025 | *f__Streptococcaceae* | *g__Streptococcus* |
| OTU555 | 4.789 | *f__Lachnospiraceae* | *g__unclassified* |
| OTU123 | 4.519 | *f__Coriobacteriaceae* | *g__Olsenella* |
| OTU224 | 4.403 | *f__Streptococcaceae* | *g__Streptococcus* |
| OTU670 | 3.804 | *f__Peptococcaceae* | *g__Peptococcus* |
| OTU1185 | 3.538 | *f__Lachnospiraceae* | *g__[Eubacterium]_rectale_group* |
| OTU931 | 3.442 | *f__Lachnospiraceae* | *g__Marvinbryantia* |
| OTU738 | 3.319 | *f__Lachnospiraceae* | *g__Oribacterium* |
| OTU10 | 3.310 | *f__Prevotellaceae* | *g__Prevotellaceae_NK3B31_group* |
| OTU684 | 3.078 | *f__Lactobacillaceae* | *g__Lactobacillus* |
| OTU1094 | 3.076 | *f__Ruminococcaceae* | *g__Faecalibacterium* |
| OTU403 | 3.034 | *f__Lachnospiraceae* | *g__Blautia* |
| OTU611 | 3.004 | *f__Streptococcaceae* | *g__Streptococcus* |
| OTU398 | 2.997 | *f__Enterobacteriaceae* | *g__Escherichia-Shigella* |
| OTU399 | 2.990 | *f__Lachnospiraceae* | *g__Oribacterium* |
| OTU1355 | 2.987 | *f__Ruminococcaceae* | *g__Oscillospira* |
| OTU928 | 2.916 | *f__Lachnospiraceae* | *g__Coprococcus_2* |
| OTU826 | 2.910 | *f__Erysipelotrichaceae* | *g__Erysipelotrichaceae_UCG-003* |
| OTU458 | 2.845 | *f__Lachnospiraceae* | *g__Lachnoclostridium* |

| OTUs | Relative abundance | | | | p-value |
| --- | --- | --- | --- | --- | --- |
|  | Mean (High_FE) | Sd. (High_FE) | Mean (Low_FE) | Sd. (Low_FE) |  |
| OTU509 | 12.120 | 6.778 | 4.563 | 2.177 | 2.45E-07 |
| OTU1013 | 0.113 | 0.0567 | 0.0388 | 0.0202 | 6.89E-08 |
| OTU197 | 0.0527 | 0.0368 | 0.0218 | 0.0163 | 3.84E-04 |
| OTU555 | 0.0405 | 0.0333 | 0.0167 | 0.0142 | 9.04E-04 |
| OTU224 | 0.0374 | 0.0342 | 0.0131 | 0.0127 | 9.07E-04 |
| OTU10 | 0.0527 | 0.00156 | 0.0065 | 0.00799 | 1.01E-03 |
| OTU398 | 0.507 | 0.503 | 0.228 | 0.273 | 8.46E-04 |

**Table S4** The OTUs that were enricher in high feed efficiency pigs within the top 24 important OTU

**Table S5** The pathways with different enrichment between high- and low-FE groups

|  | HighFE.1 | HighFE.2 | HighFE.3 | LowFE.1 | LowFE.2 | LowFE.3 |
| --- | --- | --- | --- | --- | --- | --- |
| ko00071 | 21060.6 | 20757.46 | 21027.99 | 23133.42 | 22129.99 | 22192.52 |
| ko00511 | 51609.97 | 47401.86 | 43691.56 | 41756.99 | 37609.1 | 39541.72 |
| ko00600 | 39125.22 | 37563.82 | 33578.12 | 32014.1 | 29612.17 | 31242.81 |
| ko00791 | 882.0558 | 910.413 | 888.2242 | 702.3226 | 629.9621 | 511.1081 |
| ko00903 | 1525.408 | 1725.519 | 1624.185 | 2105.622 | 2204.44 | 2012.308 |
| ko02030 | 21484.25 | 21221.47 | 22409.78 | 15520.36 | 18492.17 | 15319.51 |
| ko02040 | 24895.19 | 24608.66 | 26515.55 | 20701.31 | 22167.09 | 19546.54 |
| ko03010 | 189906.1 | 176586.5 | 188473.2 | 205931.1 | 198489 | 202527.6 |
| ko03020 | 31598.36 | 30660.92 | 31288.54 | 33925.3 | 32394.84 | 35018.07 |
| ko03320 | 19041.41 | 19829.9 | 20208.22 | 21017.51 | 21255.75 | 20596.63 |
| ko04152 | 9338.454 | 9680.329 | 10502.64 | 11122.54 | 11725.93 | 12077.4 |
| ko04210 | 3563.018 | 3439.966 | 3209.979 | 2895.364 | 2637.098 | 2452.783 |
| ko04212 | 23670.02 | 22070.87 | 22741.62 | 24327.78 | 24507.19 | 25782.21 |
| ko04622 | 147.2613 | 167.794 | 211.6955 | 270.2621 | 252.8157 | 237.5 |
| ko04924 | 47.78427 | 66.02771 | 80.43431 | 133.9567 | 105.5357 | 143.0871 |
| ko04974 | 7427.888 | 6111.35 | 5947.311 | 4950.638 | 5059.613 | 5145.736 |
| ko05133 | 3823.128 | 2673.755 | 2557.634 | 1691.939 | 1707.559 | 1710.18 |
| ko05410 | 47.78427 | 57.72894 | 80.43431 | 128.531 | 105.5357 | 143.0871 |

| Taxa | Mean (High_FE) | Variance (High_FE) | standard error (High_FE) | Mean (Low_FE) | Variance (Low_FE) | standard error (Low_FE) | p-value |
| --- | --- | --- | --- | --- | --- | --- | --- |
| endo-&beta_-1,4-xylanase (EC 3.2.1.8) | 0.001285 | 1.31E-10 | 6.61E-06 | 0.001143 | 3.23E-10 | 1.04E-05 | 0.00022 |
| endo-&beta_-1,6-galactanase (EC 3.2.1.164) | 0.000850 | 2.28E-10 | 8.72E-06 | 0.000770 | 2.5E-10 | 9.12E-06 | 0.00339 |
| arabinoxylan-specific endo-&beta_-1,4-xylanase (EC 3.2.1.-) | 0.000715 | 1.36E-10 | 6.73E-06 | 0.000632 | 4.18E-10 | 1.18E-05 | 0.00399 |
| cellulose &beta_-1,4-cellobiosidase (EC 3.2.1.91) | 0.000715 | 1.36E-10 | 6.73E-06 | 0.000632 | 4.18E-10 | 1.18E-05 | 0.00399 |
| endo-&beta_-1,4-glucanase _ cellulase (EC 3.2.1.4) | 0.000715 | 1.36E-10 | 6.73E-06 | 0.000632 | 4.18E-10 | 1.18E-05 | 0.00399 |
| endoglycoceramidase (EC 3.2.1.123) | 0.000715 | 1.36E-10 | 6.73E-06 | 0.000632 | 4.18E-10 | 1.18E-05 | 0.00399 |
| glucan &beta_-1,3-glucosidase (EC 3.2.1.58) | 0.000715 | 1.36E-10 | 6.73E-06 | 0.000632 | 4.18E-10 | 1.18E-05 | 0.00399 |
| glucan endo-1,6-&beta_-glucosidase (EC 3.2.1.75) | 0.000715 | 1.36E-10 | 6.73E-06 | 0.000632 | 4.18E-10 | 1.18E-05 | 0.00399 |
| hesperidin 6-O-&alpha_-L-rhamnosyl-&beta_-glucosidase (EC 3.2.1.168) | 0.000715 | 1.36E-10 | 6.73E-06 | 0.000632 | 4.18E-10 | 1.18E-05 | 0.00399 |
| mannan endo-&beta_-1,4-mannosidase (EC 3.2.1.78) | 0.000715 | 1.36E-10 | 6.73E-06 | 0.000632 | 4.18E-10 | 1.18E-05 | 0.00399 |
| mannan transglycosylase (EC 2.4.1.-) | 0.000715 | 1.36E-10 | 6.73E-06 | 0.000632 | 4.18E-10 | 1.18E-05 | 0.00399 |
| xyloglucan-specific endo-&beta_-1,4-glucanase (EC 3.2.1.151) | 0.000715 | 1.36E-10 | 6.73E-06 | 0.000632 | 4.18E-10 | 1.18E-05 | 0.00399 |
| exo-&beta_-1,4-galactanase (EC 3.2.1.-) | 0.000295 | 1.32E-10 | 6.64E-06 | 0.000213 | 2.71E-10 | 9.5E-06 | 0.00276 |
| rhamnogalacturonan lyase (EC 4.2.2.-) | 0.000177 | 5.71E-11 | 4.36E-06 | 0.000113 | 3.47E-11 | 3.4E-06 | 0.000411 |
| exo-unsaturated rhamnogalacturonan lyase (EC 4.2.2.-) | 0.000176 | 5.58E-11 | 4.31E-06 | 0.000111 | 4.51E-11 | 3.88E-06 | 0.000436 |
| galactoside &alpha_-1,3-L-fucosyltransferase (EC 2.4.1.152) | 5.47E-06 | 1.69E-13 | 2.38E-07 | 0.00000830 | 1.5E-13 | 2.23E-07 | 0.000655 |
| galactoside &alpha_-1,3_1,4-L-fucosyltransferase (EC 2.4.1.65) | 5.47E-06 | 1.69E-13 | 2.38E-07 | 0.00000830 | 1.5E-13 | 2.23E-07 | 0.000655 |
| glycoprotein &alpha_-1,3-L-fucosyltransferase (EC 2.4.1.214) | 5.47E-06 | 1.69E-13 | 2.38E-07 | 0.00000830 | 1.5E-13 | 2.23E-07 | 0.000655 |

**Table S6** The different EC activity abundance between the high- and low-FE groups
